# VEGF-Mediated Proliferation of Human Adipose Tissue-Derived Stem Cells

**DOI:** 10.1371/journal.pone.0073673

**Published:** 2013-10-03

**Authors:** Guangfeng Chen, Xiujuan Shi, Chen Sun, Min Li, Qing Zhou, Chen Zhang, Jun Huang, Yu Qiu, Xiangyi Wen, Yan Zhang, Yushan Zhang, Shuzhang Yang, Lixia Lu, Jieping Zhang, Qionglan Yuan, Jianwei Lu, Guotong Xu, Yunyun Xue, Zibing Jin, Cizhong Jiang, Ming Ying, Xiaoqing Liu

**Affiliations:** 1 Tenth People's Hospital, Tongji University School of Medicine, Shanghai, China; 2 Shanghai Key Laboratory of Signaling and Disease Research, School of Life Sciences and Technology, Tongji University, Shanghai, China; 3 Division of Ophthalmic Genetics, The Eye Hospital of Wenzhou Medical College and Lab for Stem Cell & Retinal Regeneration, School of Ophthalmology & Optometry, Wenzhou Medical College, Wenzhou, China; 4 Shenzhen Key Laboratory of Marine Bioresources and Ecology, College of Life Sciences, Shenzhen University, Shenzhen, China; Baylor College of Medicine, United States of America

## Abstract

Human adipose tissue-derived stem cells (ADSCs) are an attractive multipotent stem cell source with therapeutic applicability across diverse fields for the repair and regeneration of acute and chronically damaged tissues. In recent years, there has been increasing interest in ADSC for tissue engineering applications. However, the mechanisms underlying the regulation of ADSC proliferation are not fully understood. Here we show that 47 transcripts are up-regulated while 23 are down-regulated in ADSC compared to terminally differentiated cells based on global mRNA profiling and microRNA profiling. Among the up-regulated genes, the expression of vascular endothelial growth factor (VEGF) is fine-tuned by miR-199a-5p. Further investigation indicates that VEGF accelerates ADSC proliferation whereas the multipotency of ADSC remains stable in terms of adipogenic, chondrogenic and osteogenic potentials after VEGF treatment, suggesting that VEGF may serve as an excellent supplement for accelerating ADSC proliferation during *in vitro* expansion.

## Introduction

Stem cells are characterized by their ability to undergo self-renewal and multilineage differentiation and form terminally differentiated cells [1]. Ideally, stem cells for regenerative medicinal applications should meet the following set of criteria: (i) should be found in abundant quantities (millions to billions of cells); (ii) can be collected and harvested by a minimally invasive procedure; (iii) can be differentiated along multiple cell lineage pathways in a reproducible manner, and (iv) can be safely and effectively transplanted to either an autologous or allogeneic host [2]. Bone marrow-derived mesenchymal stem cells (MSC) are considered as a benchmark for adult stem cell research [3], [4]. Bone marrow derived MSCs are promising tools for basic research and regenerative medicine [5]; however, their isolation is an invasive and painful procedure that often results in a relatively low yield [6].

Recent studies have identified MSCs with similar properties in almost all mammalian tissues, suggesting that MSCs with similar clinical potential might be more readily isolated elsewhere. Arguably, one of the most promising of these MSCs is adipose tissue stem cells (ADSCs) compared with bone marrow-derived MSCs and umbilical cord blood derived MSCs [7]–[10]. ADSCs have been variously termed as pre-adipocytes, stromal cells, processed lipoaspirate cells, adipose-derived stem cells, and multipotent adipose-derived stem cells [11]. ADSCs possess many of the traits common to bone marrow-derived MSCs, including plasticity and a high proliferative potential [12]. Similar proliferation rates and gene expression pathways for MSCs and ADSCs have been documented in terms of osteogenic, chondrogenic, adipogenic and neurogenic potentials [13]–[15]. In addition to their multipotency, ADSCs can be easily isolated from readily available white adipose tissues [6], [16]. An abundant number of human ADSCs can be derived from lipoaspirate, the waste product of liposuction surgery. Processing 300 mL of lipoaspirate routinely yields 1×10^7^ to 6×10^8^ ADSCs. Moreover, they can also be cultured for longer than bone marrow-derived MSC before becoming senescent [17]–[19]. Due to the combination of these beneficial properties, ADSC research has gained more and more attention over the past decades, with particular focus on tissue engineering and regenerative medicine.

MicroRNA has emerged as a new dimension of gene regulation in recent years. An extensive number of studies have identified a plethora of noncoding RNAs that regulate diverse sets of biological processes including cell proliferation and suppression of apoptosis, oncogenesis, viral disease, and hematopoietic differentiation [20]–[22]. Noncoding RNAs bind to the 3′-untranslated region of their target genes, including key transcription factors, receptors and kinases, and regulate protein translation or mRNA stability [23], [24]. The mechanisms underlying the regulation of proliferation or multipotency of ADSCs through specific microRNAs and/or their target genes have not been thoroughly investigated and consequently are not well characterized.

In the present study, the global expression profiling of both mRNA levels and miRNA levels has revealed a subset of genes that might be involved in proliferation or multipotency of ADSC. Moreover, we have found that vascular endothelial growth factor (VEGF), a growth factor critical for angiogenesis, may be regulated by specific microRNAs. Our gain- or loss-of-function experiments have demonstrated that miR-199a-5p acts as an endogenous regulator for VEGF expression through binding to the VEGF 3′-UTR. Importantly, miR-199a-5p overexpression restrains ADSC proliferation. Our findings suggest that miR-199a-5p may act as a VEGF regulator to antagonize ADSC proliferation.

## Materials and Methods

### Cell culture

Human umbilical vein endothelial cells (HUVEC) and human dermal fibroblasts (HDF) cells were purchased from PromoCell (Heidelberg, Germany). HUVEC and HDF cells were cultured in DMEM/F12 medium supplemented with 10% fetal bovine serum(FBS), heparin (90 µg/mL), L-glutamine (2 mM), penicillin G (50 U/mL) and streptomycin sulphate (50 µg/mL). ECGS (20 µg/mL) was added to HUVEC culture medium. Cells were kept in an incubator (5% CO_2_ in humidified atmosphere). ASDCs were isolated from adipose tissues obtained from patients undergoing tumescent liposuction according to procedures approved by the Ethics Committee at the Chinese Academy of Medical Sciences and Peking Union Medical College. All patients provided written informed consent. All patients were females ranging in age from 29 to 37 years. Briefly, adipose tissue obtained from the patients was washed 3 times by phosphate-buffered saline (PBS) with 1% penicillin/streptomycin and then carefully minced by sterile operation scissors. The minced tissues were then enzymatically dissociated for 45 minutes at 37°C by 0.15% collagenase type I (GIBCO). The suspension was then neutralized with isometric culture medium and centrifuged at 500×g for 5 minutes. The cell pellet was resuspended in DMEM/F12 medium (GIBCO) supplemented with 10% FBS (GIBCO), 10% KSR (Invitrogen) or ADSC serum-free media (Biowit Technologies Ltd, Quincy, USA) at a density of 2×10^6^ cell/mL. Cell cultures were maintained at 37°C in a humidified incubator supplemented with 5% CO_2_. Passage 3 cells were used for the following experiments.

### RNA isolation and clean up

Total RNA was isolated from cell lysates from six patients using a standard phenol/chloroform extraction protocol and was stored at −80°C. MicroRNAs were isolated by using miRNeasy kit (Qiagen). Control RNAs were isolated from HUVEC and human fibroblast cells. For preparation of samples for microarray analysis, an equal volume from each of five replicate lysates per shear stress experiment was pooled prior to RNA isolation. These samples were then DNase treated using the RNase-Free DNase Set (Qiagen, West Sussex, UK) and cleaned using RNeasy MinElute Cleanup Kits (Qiagen). Total RNA concentration was assessed using Nanodrop (Thermo Fisher).

### Gene expression and microRNA profiling analysis

RNA labeling reactions and hybridizations were performed according to the manufacturer's protocol (Agilent One-Color Microarray-Based Gene Expression Analysis, Version 5.0.1). Briefly, polyA(+) RNA in 500 ng of total RNA was primed with an oligo (d)T-T7 primer and converted into dsDNA with MMLV-RT, then transcribed and simultaneously labeled with Cyanine 3-CTP for 2 hours at 40°C using the Agilent Low RNA Input Linear Amplification Kit (p/n 5188–5339). After labeling and purification, cRNA was quantified and the specific dye incorporation activity was validated using the NanoDrop ND-1000. 1.65 µg of labeled cRNA was mixed with Agilent 10× Blocking Agent and 25× Fragmentation Buffer, then incubated at 60°C for 30 hours. After fragmentation, the cRNA mixtures were immediately mixed with Agilent 2× Hybridization Buffer (p/n 5188–5339) and applied to the Agilent Human 4×44 K whole genome microarrays (G4112F) for 17 hours at 65°C. Array slides were washed with Agilent Gene Expression Wash Buffer 1 and 2 (p/n 5188–5327) and then scanned using the Agilent DNA Microarray scanner with 5 µm resolution and the eXtended Dynamic range setting (XDR Hi 100%, Low 10%) to avoid saturated features.

For mRNA microarray analysis, raw data were preprocessed with Affymetrix Microarray Suite (MAS) 5.0 software and the subsequent CHP file data were then analyzed using GeneSpring GX 7.3.1 software (Agilent Technologies, West Lothian, UK). For normalization, all signals below 0.01 were reset to 0.01. The signal for each gene was divided by the median signal of all genes from the same GeneChip. The signal from each gene in each shear stress sample was divided by the average signal for that gene in the static control sample. Comparison of ADSCs, endothelial and fibroblast cells were completed using duplicate array data sets for each cell line. The whole genomic mRNA data are available at the National Center for Biotechnology Information (NCBI) GEO database under the platform (accession number GSE48220).

MicroRNA expression analyses were performed by using Exqion mercury LNA™ (Exqion Inc). Labeling and hybridization of total RNA samples were performed according to the manufacturer's protocol. 100 ng total RNA was used as input into the labeling reaction, and the entire reaction was hybridized to the array for 20 hours at 55°C. For the microarray versus qRT-PCR comparisons, the labeling and hybridizations of the six ADSCs, human endothelial and human fibroblast cells were performed 4 to 5 times, and the means for each microRNA were calculated. The microRNA array data are available at the National Center for Biotechnology Information (NCBI) GEO database under the platform (accession number GSE48227).

### Cell proliferation

Cell proliferation was assessed by both 3-(4,5-Dimethylthiazol-2-yl)-2,5-diphenyltetrazolium bromide (MTT) assay and trypan blue assay. For MTT assay, ADSCs (2×10^4^ cells/well) were seeded in 48-well plates. After 24 hours, 20 µl MTT (5 mg/ml) was added and incubated for 4 hours at 37°C; the reaction was stopped by replacing the MTT-containing medium with 100 µl DMSO and the formazan salts were dissolved by gentle shaking for about 10 minutes at room temperature. For colorimetric analysis, the absorbance at 490 nm was recorded using a microplate reader (Bio-Rad 680, Bio-rad, USA). For trypan blue assay, Cell number was counted using trypan blue exclusion viable cell assay. Briefly, cells were trypsinized and resuspended in equal volumes of medium and trypan blue (0.05% solution) and counted using a haemocytometer. Trypan blue dye (Invitrogen) exclusion was used to assess cell viability. Each assay was repeated at least three times.

### Gene expression and silencing, viral production, infection and efficiency in ADSCs

Short hairpin RNA (shRNA) for the VEGF gene silencing in an adeno-associated viral vector (AAV) were constructed and packaged into viral particles with a titer of over 1×10^12^/ml by BioWit Technologies Ltd. Lentiviral vector harboring microRNAs were constructed into pLVX-ShRNA2 vector and particles were produced by using 2-generation packaging mix (BioWit Technologies Ltd.) with over a titer of over 1×10^8^ TU. For lentivirus infection, ADSCs were grown to 70 to 80% confluence and infected with VEGF shRNA lentivirus or control lentivirus harboring a scrambled miRNA at a multiplicity of infection (MOI) of 10. To determine the infection efficiency, cells expressing ZsGreen protein were observed using fluorescence microscopy (IX71, Olympus, Tokyo, Japan) two days after infection. To investigate the effects of miR-199a-5p inhibition, ADSCs were seeded at 5×10^4^ cells/well in 6 well plates and transfected with commercial synthetic miR-199a-5p inhibitor or inhibitor negative control (RIBOBIO co., Ltd, Guangzhou, China) at a final concentration of 300 nM using lipofectamine 2000 (Invitrogen).

### Quantitative real time (qRT)-PCR analysis

Total RNA was extracted using Trizol reagent (Invitrogen) and microRNA was isolated using miRNeasy kit (Qiagen) according to the manufacturer's instructions. RNA (10 ng) was reverse-transcribed using MultiScribe reverse transcriptase, reverse transcriptase buffer, dNTPs, RNase inhibitor and miR-specific primers in the GeneAmp 9700 PCR system (all from Applied Biosystems, Foster City, CA, USA). The cDNA obtained was used for real-time (RT) PCR using miR-specific TaqMan primers (Applied Biosystems, Foster City, CA, USA). For relative quantification, the expression of U6snRNA (Applied Biosystems) was used as an endogenous control. To quantify mRNA, 1 µg total RNA was reverse-transcribed using a Revert Aid^™^ first-strand cDNA synthesis kit (Fermentas, Burlington Ontario, Canada). Subsequently, the mRNA expression of the target gene was determined by SYBR Green assays (Bio-Rad, Hercules, CA, USA). SYBR Green QPCR SuperMix-UDG was purchased from Invitrogen. Quantitative PCR was performed using an Applied BioSystems 7300 sequence detection system. All experiments were performed in triplicate. The level of expression was calculated based on the PCR cycle number, and the relative gene expression level was determined using the ΔΔ*C*_t_ method[25]. The primers for VEGF qPCR were V5 (5′GGCAGAATCACGAAGTGGTG3′) and V3 (5′GGGTCTCGATTGGATGGCAGTAG3′). The primers for GAPDH qPCR internal control were G5 (5′CTCTCTGCTCCTCCTGTTCGAC3′) and G3 (5′TGAGCGATGTGGCTCGGCT3′).

### Enzyme-linked immunosorbent assay (ELISA)

Immunoreactive VEGF protein levels were measured using commercial Enzyme-linked immunosorbent assay (ELISA) tests in accordance with the manufacturer's instructions (ExCell Biology, Inc., Shanghai, China). Cell growth medium was collected 36 hours after infection, and the cells were harvested and counted. Supernatants were then centrifuged at 14000× g for 5 min to remove cellular debris, and subsequently stored at −70°C until needed. All standards and supernatants from experimental and control cultures were assayed in triplicate and the values were averaged.

### Flow cytometry

Flow cytometry was performed for cell lineage classification prior to differentiation. The following fluorochrome-labelled monoclonal antibodies were used: FITC-CD29 (R&D Systems), and FITC-CD44 (R&D Systems). To analyze the control samples, different immunoglobulin (Ig) G isotypes coupled to FITC (R&D system) were used. The cells were suspended in PBS with 2% FBS and were incubated for 45 minutes at 4°C in the dark. Subsequently, the cells were washed twice with PBS and centrifuged for 6 minutes at 500×g, removing residual fluorochrome to avoid false positive results. Depending on cell quantity, the pellets were suspended in 300 to 600 µl of PBS with 2% FBS. All flow cytometry measurements were made using FACS Calibur (BD Biosciences).

### Immunocytochemistry

The cells were fixed with 4% paraformaldehyde for 10 minutes at room temperature and then incubated with Tween 20 for 4 minutes at room temperature to increase permeability. The slides were incubated sequentially for 1 hour each with FITC-coupled primary antibody against CD29 and CD44 (R&D system) and for 1 hour with FITC-coupled anti-mouse IgG (BD Pharmingen). Between each step, the slides were washed with PBS containing 1% BSA (Sigma). The cells were examined by fluorescence microscopy (IX71, Olympus,Tokyo).

### Adipogenic, chondrogenic and osteogenic differentiation of ADSCs

For adipogenic differentiation, ADSCs from passage 3 were trypsinized and replated at a density of 5×10^5^ cells per T25 cm^2^ flask. The cells were incubated in 3 ml DMEM/F12 medium with 10% FBS for a day and the cells were then placed in the adipogenic differentiation medium: DMEM/F12 medium containing 5% FBS, 1 µM dexamethasone (Sigma, St. Louis, USA), 10 µM insulin (Wako, Osaka), 200 µM indomethacin (Sigma, St. Louis), and 0.5 mM isobuthyl-methylxanthine (Sigma, St. Louis). The media were changed every 3 days for two to three weeks. Adipogenic differentiation was assessed by Oil Red O staining. For Oil Red O staining, the cells were assessed using an Oil Red O stain as an indicator of intracellular lipid accumulation. Prior to staining, the cells were rinsed with PBS and fixed in 4% formaldehyde for 30 minutes. They were then incubated in 2% (wt/vol) Oil Red O reagent for 30 minutes at room temperature. Excessive stain was removed by washing with PBS for 3 times.

For chondrogenic differentiation, ADSCs from passage 3 were trypsinized and replated at a density of 1×10^5^ cells per T25 cm^2^ flask. The cells were incubated in 3 ml DMEM/F12 with 10% FBS for a day and the medium was then placed in the chondrogenic differentiation medium DMEM/F12 containing: 1% insulin-transferring-selenium (Sigma), 5% FBS, 5 mg/mL linoleic acid (Sigma), 50 mg/ml ascorbate-2-phosphate (Sigma), 1% penicillin-streptomycin (Gibco), and 10 ng/mL TGF-β3 (Sigma). The medium was changed every 3 days for two to three weeks. Chondrogenic differentiation was assessed by Alcian blue staining. For Alcian blue staining, cultures were rinsed twice with PBS, fixed in 4% (w/v) paraformaldehyde for 15 minutes, and incubated in 1% (w/v) Alcian blue 8-GX (Sigma) for 3 hours at room temperature. Excessive stain was removed by washing with PBS 3 times.

For osteogenic differentiation, ADSCs from passage 3 were trypsinized and replated at a density of 5×10^5^ cells per T25 cm^2^ flask. The cells were incubated in 3 ml DMEM/F12 with 10% FBS for 1 day and the medium was then placed in the osteogenic differentiation medium. DMEM/F12 medium contained: 5% FBS, 1 µM dexamethasone, 20 mM b-glycerolphosphate and 50 mM ascorbate 2-phosphate. The medium was changed every 3 days for two to three weeks. For alizarin red staining, the cells were rinsed twice with PBS and fixed in 4% (w/v) paraformaldehyde for 15 minutes, and incubated in 1% (w/v) alizarin red for 3 to 5 minutes at room temperature. Excessive stain was removed by washing with PBS 3 times.

### Luciferase assay

ADSCs were infected with lentiviral expressing miR-199a-5p and then seeded in 48-well plates with 2×10^4^ cells per well. Briefly, the cells were then transfected with 2 µg of pLuci vector or pLuci-3′UTR mut vector or pLuci-3′UTR. The pRL-TK containing Renilla luciferase was also cotransfected as a reference control. Luciferase activities were measured 24 hours after transfection by using dual-luciferase reporter assay (Promega). The firefly luciferase activity was normalized to Renilla luciferase activity. The pLuci-3′UTR mut vector for the miR-199a-5p binding site of VEGF 3′UTR was constructed by using GeneTailor site-directed mutagenesis system (Invitrogen). A lentiviral vector with a putative microRNA 21-recognition element (single copy) from the VEGF gene fused with the firefly luciferase gene as a 3′-untranslated region was provided by BioWit Technologies Ltd. All experiments were repeated three times.

### Statistical analysis

Data are shown as the mean ± SEM, The statistical significance of the difference between two groups was evaluated with the Wilcoxon Signed-rank test.

## Results

### Comparative genome-wide gene and microRNA expression profiles

**Figure 1:**
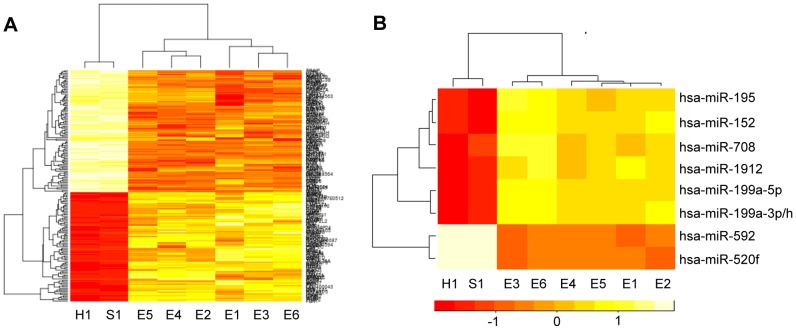
Global expression profiles of mRNAs and microRNAs. (A) Hot map of mRNA levels. (B) Hot map of miRNA levels. A set of mRNAs and miRNA were significantly differentially expressed in human endothelial cells, human fibrobast cells and ADSCs. Red color indicated high expression whereas yellow color indicated low expression. H1, human endothelial cell; S1, human fibroblast cell; E1–E6, human adipose tissue-derived stem cells (ADSCs).

Multipotency is the significant different characteristic of ADSCs compared with many other terminally differentiated cells such as human endothelial cells and fibroblast cells. In an effort to reveal the functions essential for this process, we used microarray (Agilent 4×44 K Whole Human Genome Microarray Kit) to profile the global gene expression in ADSCs by including human endothelial and fibroblast cells as controls. Clustering analysis revealed a set of genes sharing a highly similar level of expression between human endothelial and fibroblast cells, whereas ADSCs had a significantly different expression profile compared to human endothelial and fibroblast cells (Fig. 1A). Similarly, we profiled the global microRNA expression in ADSCs, fibroblasts and endothelial cells by using Exqion mercury LNA™ (Exqion Inc) (Fig. 1B). Interestingly, we only identified six microRNAs significantly up-regulated in ADSCs and two microRNAs with a reverse expression pattern in the fibroblasts and endothelial cells (Table 1 and Table 2).

**Table 1:** Up-regulated microRNAs.

ID	Name	P Value	Fold Change
10995	hsa-miR-199a-3p/has-miR-199b-5p	0.05	2.74
13148	hsa-miR-195-	3.85e-06	2.18
17676	hsa-miR-152	0.02	2.32
29190	hsa-miR-708	0.05	2.00
29562	hsa-miR-199a-5p	0.01	2.21
146168	hsa-miR-1912	0.04	2.12

**Table 2:** Down-regulated microRNAs.

ID	Name	P Value	Fold Change
17312	Hsa-miR-592	0.00003	0.44
42455	Hsa-miR-520f	0.03	0.49

MicroRNA usually regulates multiple target genes, vice versa. Therefore, it is a challenge to identify the potential target genes of a given microRNA with high confidence. In order to identify reliable target genes of the above eight differentially expressed microRNAs, we first collected their target genes annotated in the two popular databases TargetScan and miRanda. Next, we obtained the intersection of the target genes from the two databases of microRNAs. Finally, we checked whether the target genes were significantly differentially expressed by gene expression profiling analysis. We only retained the target genes that were also significantly differentially expressed between ADSCs and terminally differentiated cells (fibroblast cells and endothelial cells). A total of 70 target genes of 8 microRNAs were identified (Table 3). Further bioinformatics analysis predicted that 47 transcripts were up-regulated while 23 transcripts were down-regulated in ADSC compared to terminally differentiated cells if the intersection of transcripts and miR-predicted targets was applied. We further employed literature mining to identify the genes with possible roles in regulating the proliferation or multipotency of ADSCs. Comparative analysis supported the view that VEGF might be one of the abundant genes and its expression may be regulated by miR-199a-5p.

**Table 3:** Up-regulated and down-regulated genes in both arrays.

Up-regulated genes		Down-regulated genes
ACAN	LBH	TRIM6
ARHGAP24	LBX2	ASF1B
ASPRV1	LY6K	BANK1
BST1	MGC42105	C17orf53
C10orf110	MME	C7orf46
CD163	NID2	CENPF
COL10A1	PGF	CETN3
COL5A1	PID1	CUL2
CSF1	PPFIBP2	DCLER1A
CXCR7	RAD50	EMB
EFHA2	RASD1	EZR
EGR1	RHOBTB3	FAM54A
F3	SLC5A3	FLJ40504
FBN1	SMAD7	HMGN5
GBP2	STMN2	KLHDC9
GPC6	SVEP1	KRT18
H19	TCF4	LIPT2
HAPLN1	TIAM2	MCM8
HEG1	VCAN	PDE1C
HOXB7	ZBTB43	PFAS
ID4	VLDLR	RFC3
JHDMID	YPEL2	SAMD9L
KIAA1462	VEGF	ANKRD29
KLF7		

P Value<0.01, fold change >2 or fold change <0.5.

### The effects of VEGF on ADSC proliferation

**Figure 2:**
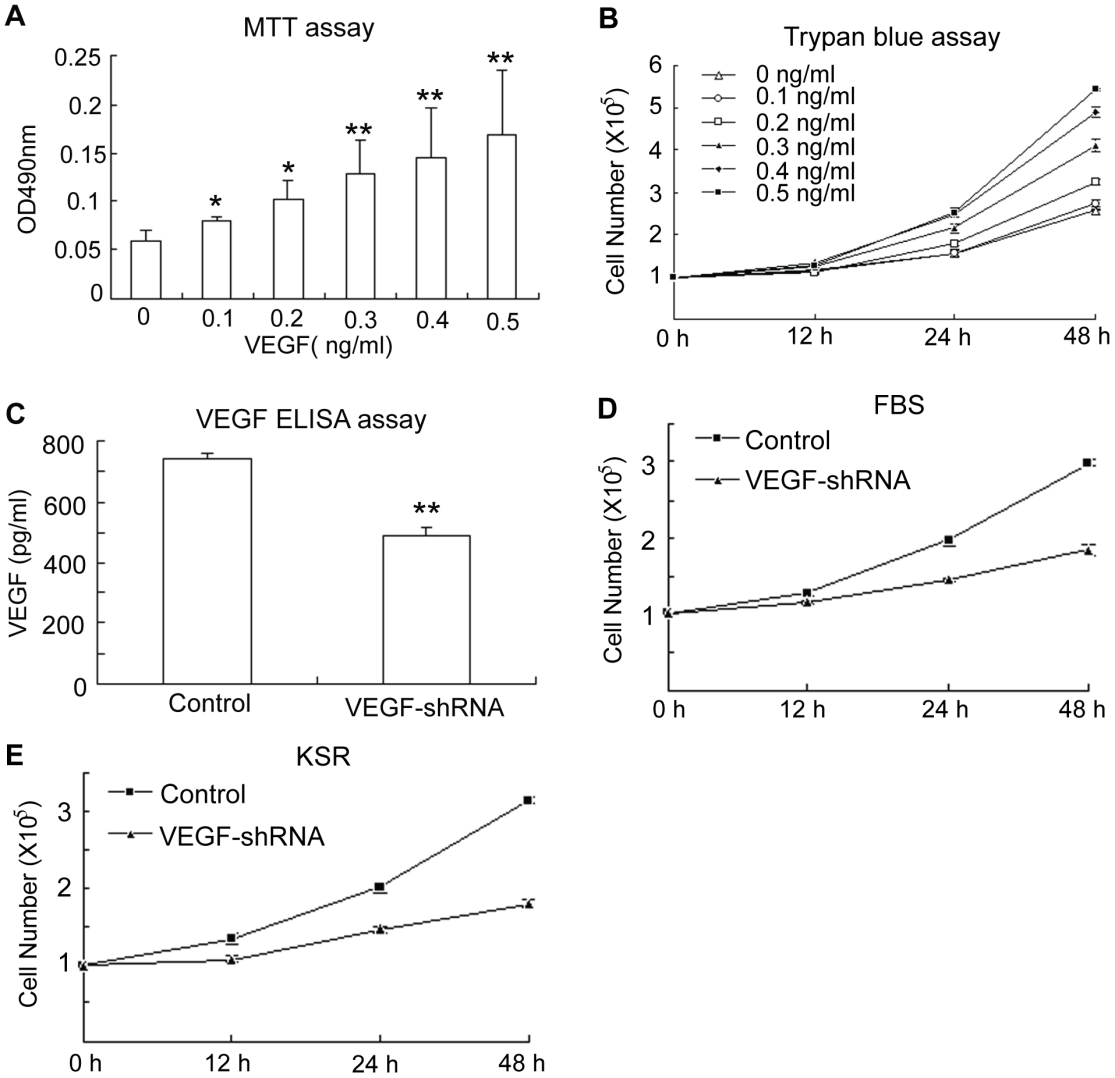
VEGF-mediated proliferation of ADSCs. (A) MTT assay indicated that proliferation of ADSCs was accelerated by supplementation of a gradient concentration of VEGF (n = 5); (B) Trypan blue staining confirmed that the effect of VEGF on ADSC proliferation was dose-dependent (n = 3, p<0.01); (C) ELISA assay indicated that VEGF expression was reduced by approximately 40% in ADSCs after shRNA silencing (n = 5, p<0.01); (D) Cell number was significantly reduced when ADSCs were cultured in FBS-containing medium for 24 or 48 hours after VEGF silencing (n = 3, P<0.01); (D) The cell number reduction was also confirmed when ADSCs were cultured for 24 or 48 hours in KSR-containing medium after VEGF silencing (n = 3, P<0.01); (E) ADSC proliferation was also evaluated in FBS medium by trypan blue assay when VEGF expression was knocked down, it was demonstrated that VEGF reduction resulted in inhibition of ADSC proliferation (n = 5). * *P*<0.05, **P<0.01.

To examine whether VEGF affected ADSC proliferation, ADSCs were cultured in media supplemented with different concentrations of exogenous VEGF (0, 0.1, 0.2, 0.3, 0.4, and 0.5 ng/ml). By both MTT and trypan blue assays, we found that VEGF supplementation promoted ADSC proliferation in a dose-dependent manner (Fig. 2A, B). We further evaluated the effects of endogenous VEGF by shRNA-mediated knockdown. Because the adeno-associated viral vector (AAV) contained the coding sequence of green fluorescent protein (ZsGreen) whose expression was driven by cytomegalovirus promoter, the efficiency of AAV infection was determined by a fluorescent microscope. Single infection with AAV showed that over 80% ADSCs expressed ZsGreen. RT-PCR indicated that VEGF was significantly silenced in ADSCs (Figure S1). To examine the effects of VEGF in regulating cell growth, we infected the cells with AAV particles expressing VEGF shRNA and subjected them to proliferation assays. Cell number counting by trypan blue assay revealed that down-regulation of VEGF reduced the ADSCs proliferation rate by approximately 38% after 48 hours (Fig. 2C, D). Of note, KSR has been reported to be more effective in making inducible multipotent stem cells than FBS [26], we also utilized KSR instead of FBS for the maintenance of ADSCs in an undifferentiated state. ZsGreen fluorescence indicated that VEGF shRNA delivery efficiency in KSR medium was also very high (Figure S1). Trypan blue assay revealed that VEGF silencing reduced the cell proliferation rate by 43%, strongly suggesting that VEGF plays a role in ADSC proliferation (Fig. 2E).

### The multipotency of cultured ADSCs after VEGF treatment

**Figure 3:**
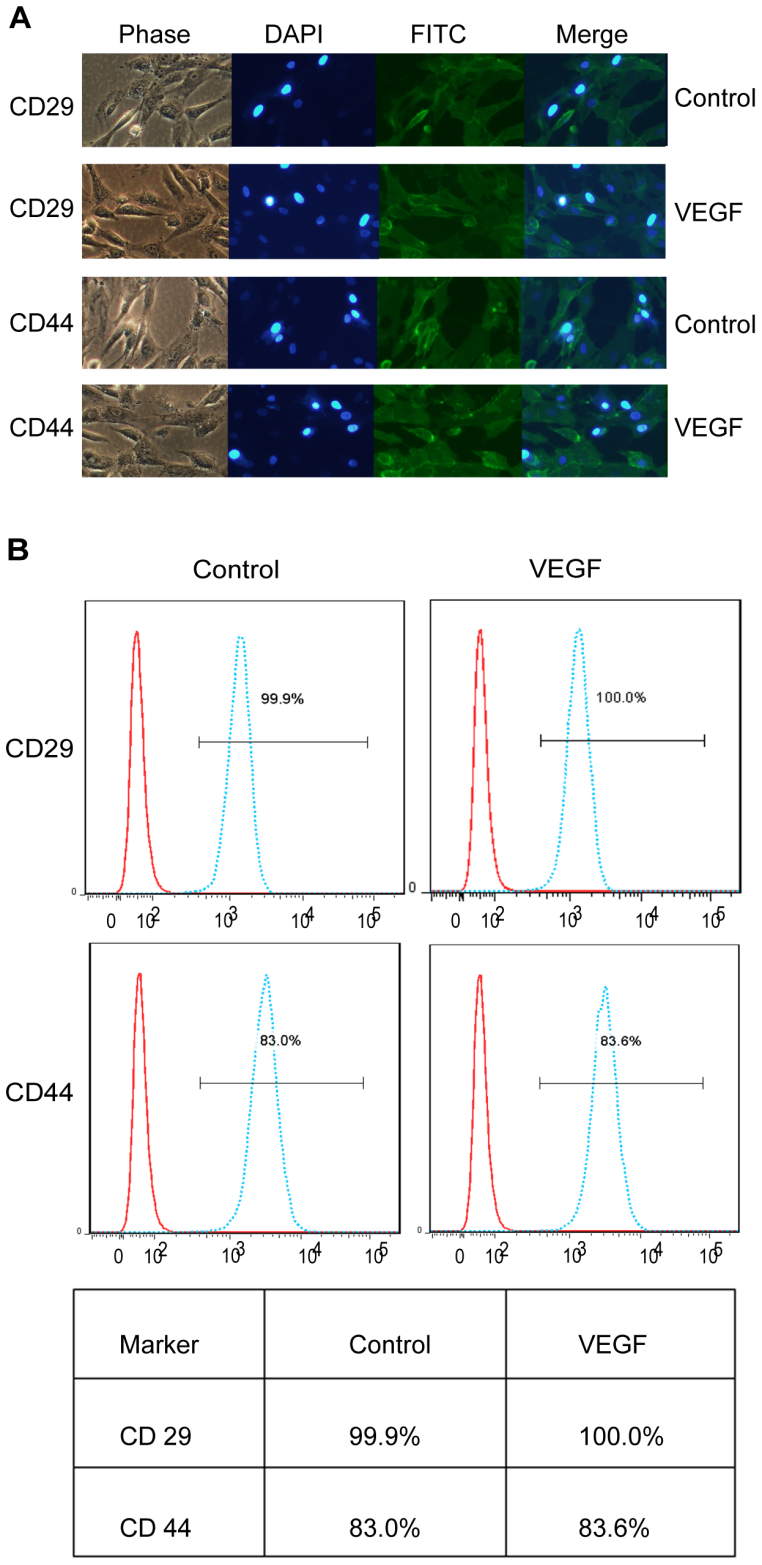
The maintenance of multipotent cell surface markers in ADSCs. (A) CD29 and CD44 staining revealed that cells had a similar pattern of expression compared to the untreated group after VEGF treatment; (B) Flow cytometry indicated that ADSC surface markers (CD29 and CD44) showed similar abundance compared with the untreated group.

To investigate whether VEGF changed the multipotency of ADSCs after proliferation, we utilized immunohistochemistry to analyze the ADSC surface markers CD29 and CD44. Immunofluorescence imaging showed a similar pattern in terms of the multipotency of cell surface markers CD29 and CD44 (Fig. 3A). Moreover, the multipotency of these cells was also confirmed by flow cytometry analysis. We found that the expression of the following markers was comparable to that of untreated controls: CD29 control (99.9%), CD29 (100.0%), CD44 control (83.0%), CD44 (83.6%) (Fig. 3B).

### The effects of VEGF on the adipogenic, chondrogenic and osteogenic differentiation of ADSCs

**Figure 4:**
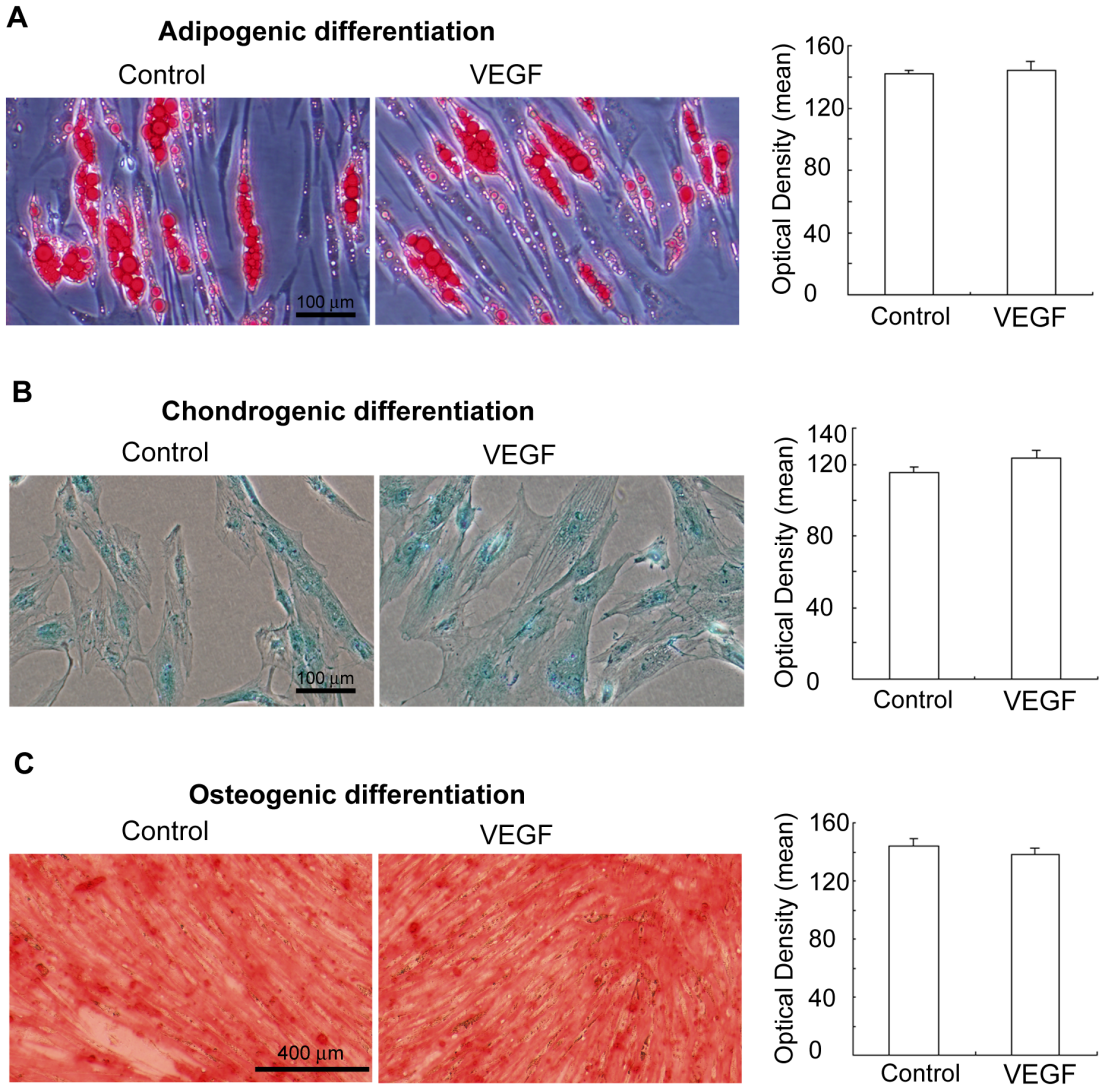
The multipotency maintenance of ADSCs after VEGF treatment. The VEGF treatment group showed similar efficiency in terms of multipotency. Relative optical intensity for adipocytes, chondrocytes and osteocytes were quantified from the images using Image-pro plus software. There was no statistic difference between the VEGF treated group and untreated group. The values were mean ± SEM (n = 3, P>0.05). (A) adipogenic potential; (B) chondrogenic potential (B); and osteogenic potential (C).

Expanded ADSCs were characterized by the expression of surface markers and the capability of differentiating into adipocytes and chondrocytes. To investigate the effects of VEGF treatment on the multipotency of ADSCs, we induced ADSCs to differentiate along adipogenic or chondrogenic lineages with the appropriate medium. For adipogenic differentiation, after primary culture in the control medium and expansion to three passages, the cells were placed into adipogenic medium. Two weeks after initial induction, we observed lipid-filled cells. The cells were stained positively for Oil Red O staining, an established lipid dye, In ADSCs there was no difference between control and VEGF treated cells (Fig. 4A). For chondrogenic differentiation, ADSCs had a spherical shape greater than 1 mm in diameter. The chondrogenic potential of ADSCs was evaluated by alcian blue staining (Fig. 4B). The chondrogenic potential of ADSCs also showed no difference following VEGF treatment. Based on alizarin red staining, cells maintained similar osteogenic potential (Fig. 4C). These observations suggest that VEGF treatment would not change the multipotency of ADSCs in terms of adipogenic, chondrogenic and osteogenic potential.

### MiR-199a-5p differentially regulates the expression of VEGF in ADSCs

**Figure 5:**
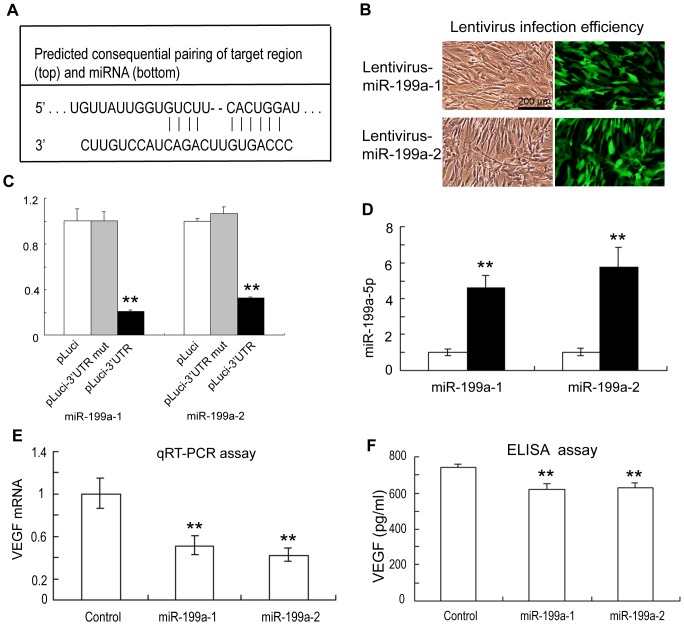
MiR-199a-5p-mediated VEGF down-regulation in ADSCs. (A) Targetscan indicates that miR-199a-5p binds the 3′UTR of VEGF mRNA; (B) Lentiviral infection efficiency was evaluated by ZsGreen intensity; (C) Dual-luciferase reporter assay indicated that miR-199a-5p interacted with the 3′UTR of VEGF mRNA by using lentiviral infection (n = 3); (D) qRT-PCR assay indicated that the lentiviruses overexpressed miR-199a-5p (n = 3); (E) qRT-PCR assay indicated that miR-199a-5p overexpression in ADSCs reduced VEGF mRNA levels by approximately 42% (n = 3). (F) ELISA assay indicated that miR-199a-5p overexpression in ADSCs reduced VEGF protein levels by approximately 16% (n = 5). The values were mean ± SEM (n = 5). **P<0.01.

To investigate the regulatory mechanisms underlying miR-199a-5p effects on VEGF expression in ADSCs, we examined the expression status of two human microRNA coding sequences, miR-199a-1 (on chromosome 19) and miR-199a-2 (on chromosome 1) that potentially contribute to miR-199a-5p transcription in ADSCs. A summary of miR-199a-5p target sites in the 3′UTRs of VEGF is presented in Fig. 5A. To verify this prediction, a standard luciferase reporter assay was conducted in ADSCs. We studied the role of endogenous miR-199a-5p in repressing VEGF expression by the dual-luciferase reporter assay. We constructed miR-199a-1 and miR-199a-2 lentiviral vectors by inserting sequences complementary to the miR-199a-5p strand. The infection efficiency of these two lentivirus overexpression miR-199a-5p was more than 90% (Fig. 5B). Luciferase activities were measured 24 hours after reporter vectors were transfected into ADSCs infected with lentivirus overexpression miR-199a-5p. The signals of *Renilla* luciferase were normalized to those of firefly luciferase. A firefly luciferase reporter vector containing mutant VEGF 3′ UTR named pLuci-3′UTR mut was used as a negative control. There was significant repression on the pLuci-3′UTR group in comparison to the pLuci-3′UTR mut group 24 hours after transfection (Fig. 5C). QRT-PCR analysis confirmed that ADSCs infected with the miR-199a-5p lentivirus exhibited higher miR-199a-5p levels (Fig. 5D). Furthermore, up-regulation of miR-199a-5p in ADSCs was also found to attenuate VEGF mRNA level by qRT-PCR and ELISA assays (Fig. 5E, F).

### MiR-199a-5p differentially modulates ADSC proliferation

**Figure 6:**
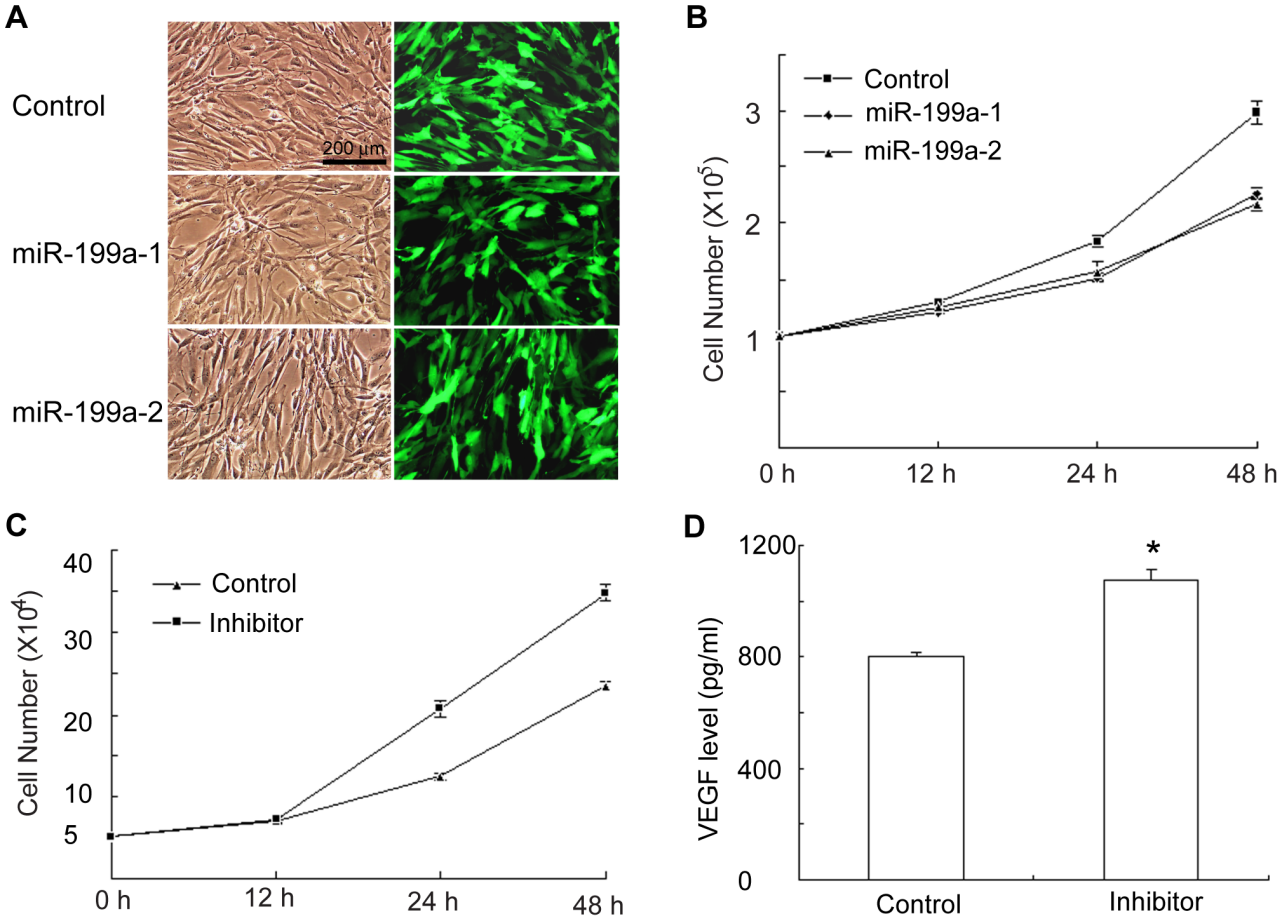
The inhibitory effect of miR-199a-5p on ADSC proliferation. (A) ZsGreen intensity indicated that most ADSCs were effectively infected by lentiruses that contained miR-199a-1, miR-199a-2 or scrambled miRNA; (B) Trypan blue assay indicated that miR-199a-5p overexpression inhibited ADSC proliferation; (C) Trypan blue assay indicated that miR-199a-5p inhibitor promoted ADSC proliferation; (D) ELISA assay revealed that miR-19-5p inhibitor increased VEGF levels in ADSCs. The values were mean ± SEM (n = 5). *P<0.05, **P<0.01.

To determine the role of miR-199a-5p in ADSC proliferation, we overexpressed miR-199a-5p in ADSCs infected with the miR-199a-5p lentivirus. Cells were divided into 3 groups as follows: scrambled miRNA (control), miR-199a-1 and miR-199a-2. Lentiviral infection efficiency was determined by detecting the expression of ZsGreen by fluorescence microscopy. At the highest infection efficiency, ZsGreen was identified in more than 90% of ADSCs (Fig. 6A). Cell number counting by trypan blue staining revealed that ADSC proliferation in the miR-199a-1 group or the miR-199a-2 group was inhibited by 24 to 27% whereas the miR-199a-5p inhibitor increased its proliferation rate by 49% (Fig. 6B, C). ELISA assay indicated that miR-199a-5p inhibitor increased VEGF levels by 38% (Fig. 6D). These observations were also consistent with MTT assays (Figure S2). Taken together, our data suggest that the inhibitory effect of miR-199a-5p on ADSC proliferation is very likely to due to down-regulation of the VEGF gene.

## Discussion

ADSCs are an attractive and abundant stem cell source with therapeutic applicability in diverse fields for the repair and regeneration of acute and chronically damaged tissues. Therefore, the safety, reproducibility and quality of ADSCs must be thoroughly examined prior to extensive use in clinical applications [27]. However, the molecular mechanisms involved in the regulation of ADSC proliferation are as yet unknown.

To better characterize the molecular basis of ADSC proliferation, gene and microRNA expression profiles of ADSCs were compared in this study. A group of genes and microRNAs whose expression differed in ADSCs compared to those in human fibroblast and endothelial cells were identified. We have further demonstrated that VEGF positively regulates ADSC proliferation *in vitro* and identified miR-199a-5p as a potent negative regulator for VEGF expression. A line of investigation has demonstrated the importance of VEGF in cell proliferation for endothelial cells and hepatocytes [28], [29]. Moreover, it has been reported that VEGF can promote neural stem cell proliferation depending on basic fibroblast growth factor via the extracellular signal-regulated kinase pathway, and overexpression of VEGF in neural stem cells further enhanced proliferation of glial progenitors [30], [31]. VEGF also increased the expression of gene products involved in anti-apoptosis and proliferation. These proteins participate in the generation of specific biological responses that are related to cellular proliferation, cell cycle progression, viability and motility. In this study, we have also demonstrated that down-regulation of VEGF by either VEGF shRNA or miR-199a-5p decreases ADSC proliferation, indicating that VEGF plays a role in ADSC proliferation.

To more thoroughly characterize the effects of VEGF in ADSCs, flow cytometry analyses were performed. Cells were assessed for the expression of commonly used MSC surface markers CD29 and CD44. After VEGF supplementation, ADSCs were still positive for CD29 and CD44. The expression patterns of surface markers were consistent with those of MSCs. In addition, VEGF treatment didn't affect ADSC multipotency as indicated by their ability to differentiate *in vitro* into a variety of cell types including adipocytes, chondrocytes and osteocytes. Of note, intracellular VEGF may regulate the balance between osteoblast and adipocyte differentiation for bone marrow MSCs in mice and it likely controls the fate of MSCs by regulating the transcription factors RUNX2 and PPARγ2 as well as through a reciprocal interaction with nuclear envelope protein lamin A/C [32]. Although VEGF may also have potential in promoting the differentiation of ADSCs into endothelial progenitor-like cells [33], our study suggests that their multipotency in confined to adipogenic, chondrogenic and osteogenic differentiation following VEGF treatment. We have also examined the effects of VEGF on human bone marrow MSCs and human umbilical cord stem cells. However, we did not find an obvious effect on proliferation (data not shown). Taken together, VEGF could promote ADSC proliferation and miR-199a-5p regulates ADSC proliferation by fine-tuning VEGF expression level. Given that VEGF may play multiple roles *in vivo*, increased VEGF secretion from ADSCs is not a desired outcome for clinical applications. Thus, targeting VEGF/miR-199a-5p signaling may serve as a strategy for tissue engineering or clinical use of ADSCs during *in vitro* expansion.

## Supporting Information

Figure S1 **The effects of VEGF on ADSC proliferation by MTT assay.** (A) The number of ADSCs was increased after VEGF treatment (0.5 ng/mL). PBS served as a control; (B) ZsGreen intensity indicated that AAV efficiently delivered VEGF shRNA into ADSCs cultured in FBS medium; (C) RT-PCR indicated that VEGF mRNA levels in ADSCs were reduced by approximately 60% when cells were cultured in FBS medium (n = 3); (D) The proliferation of ADSCs in FBS medium when VEGF expression was knocked down, suggest that down-regulation of VEGF resulted in a lower proliferation rate of ADSCs (n = 5); (E) ZsGreen intensity indicated that AAV efficiently delivered VEGF shRNA into ADSCs cultured in KSR medium; (F) The inhibitory effect on ADSC proliferation of down-regulation of VEGF was confirmed in KSR medium (n = 5). The values were mean ± SEM (n = 5). * *P*<0.05, **P<0.01.
(TIF)

Figure S2 **The inhibitory effect of miR-199a-5p on hADSCs proliferation by MTT assay.** (A) Overexpression of miR-199a-1 or miR-199a-2 by lentiviral vector significantly inhibited ADSC proliferation after culture for 24 or 48 hours; (B) miR-199a-5p inhibitor promoted ADSC proliferation after culture for 24 or 48 hours. The values were mean ± SEM (n = 5). *P<0.05, **P<0.01.
(TIF)
