# Supplementary figures and images for: VEGF-Mediated Proliferation of Human Adipose Tissue-Derived Stem Cells
Source: PLoS One. 2013 Oct 3;8(10):e73673. doi: 10.1371/journal.pone.0073673 (PMC3789739; doi:10.1371/journal.pone.0073673)

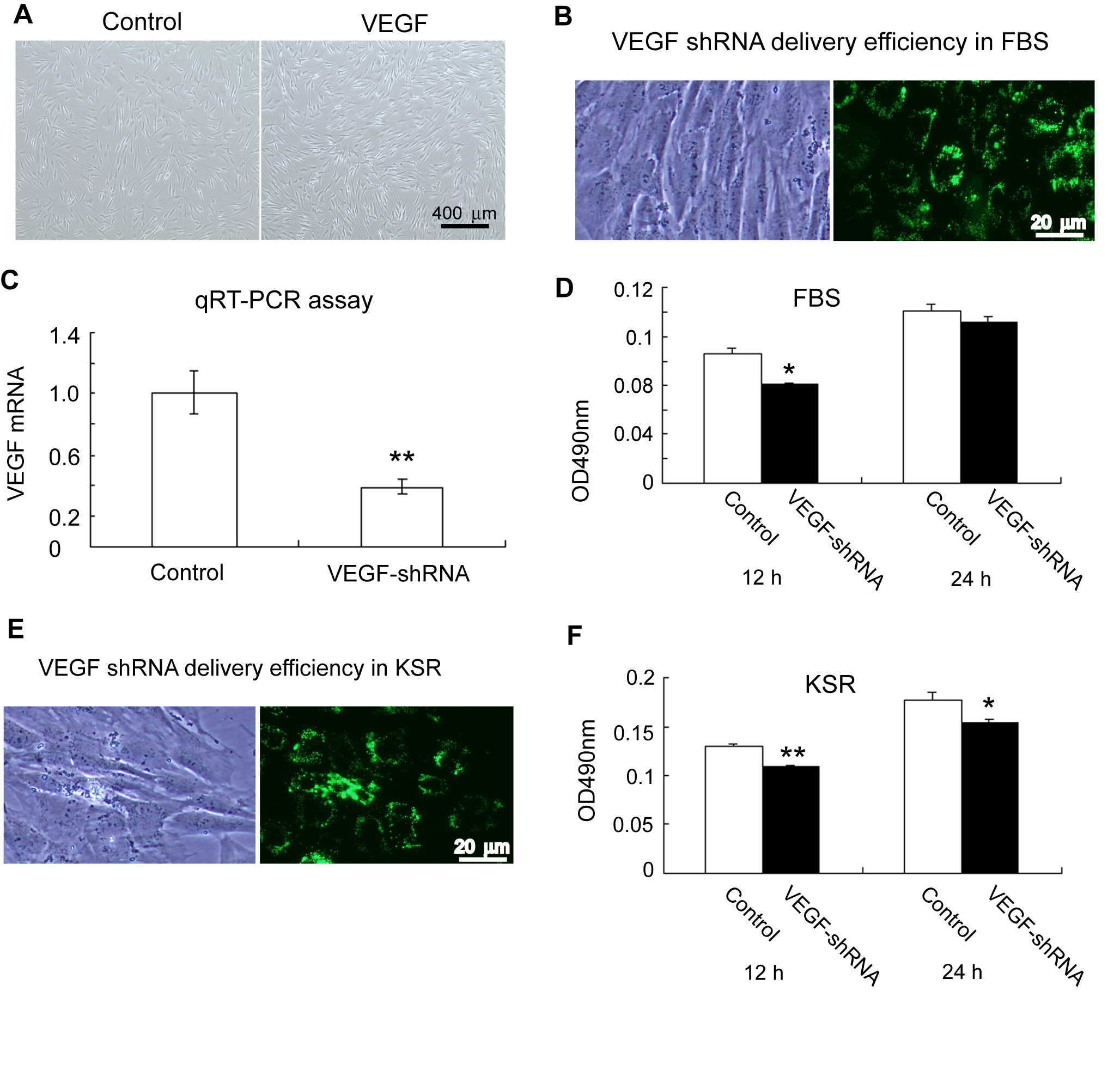

Supplement: Figure S1 — The effects of VEGF on ADSC proliferation by MTT assay. (A) The number of ADSCs was increased after VEGF treatment (0.5 ng/mL). PBS served as a control; (B) ZsGreen intensity indicated that AAV efficiently delivered VEGF shRNA into ADSCs cultured in FBS medium; (C) RT-PCR indicated that VEGF mRNA levels in ADSCs were reduced by approximately 60% when cells were cultured in FBS medium (n = 3); (D) The proliferation of ADSCs in FBS medium when VEGF expression was knocked down, suggest that down-regulation of VEGF resulted in a lower proliferation rate of ADSCs (n = 5); (E) ZsGreen intensity indicated that AAV efficiently delivered VEGF shRNA into ADSCs cultured in KSR medium; (F) The inhibitory effect on ADSC proliferation of down-regulation of VEGF was confirmed in KSR medium (n = 5). The values were mean ± SEM (n = 5). * P<0.05, **P<0.01. (TIF) [file pone.0073673.s001.tif]

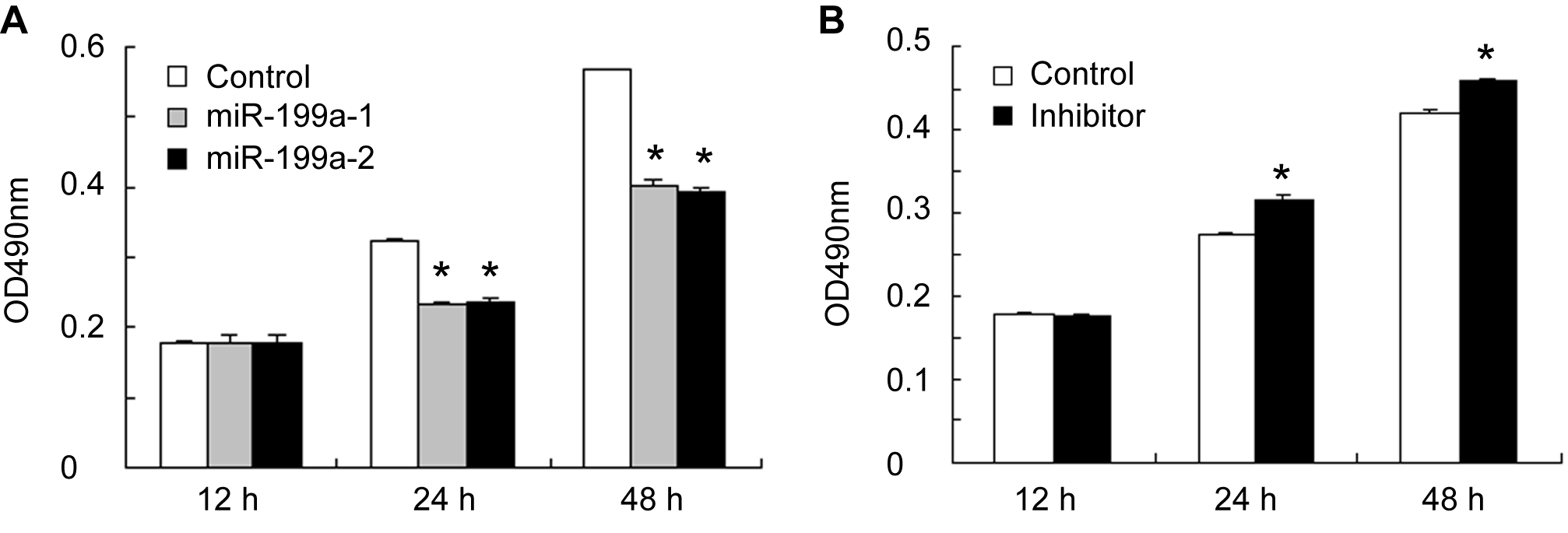

Supplement: Figure S2 — The inhibitory effect of miR-199a-5p on hADSCs proliferation by MTT assay. (A) Overexpression of miR-199a-1 or miR-199a-2 by lentiviral vector significantly inhibited ADSC proliferation after culture for 24 or 48 hours; (B) miR-199a-5p inhibitor promoted ADSC proliferation after culture for 24 or 48 hours. The values were mean ± SEM (n = 5). *P<0.05, **P<0.01. (TIF) [file pone.0073673.s002.tif]
